# Combined therapy of platelet-rich plasma and basic fibroblast growth factor using gelatin-hydrogel sheet for rotator cuff healing in rat models

**DOI:** 10.1186/s13018-021-02771-1

**Published:** 2021-10-16

**Authors:** Takeshi Kataoka, Yutaka Mifune, Atsuyuki Inui, Hanako Nishimoto, Takashi Kurosawa, Kohei Yamaura, Shintaro Mukohara, Takehiko Matsushita, Takahiro Niikura, Yasuhiko Tabata, Ryosuke Kuroda

**Affiliations:** 1 Department of Orthopaedic Surgery, Kobe University Graduate School of Medicine, 7-5-2, Kusunoki-cho, Chuo-ku, Kobe, 650-0017 Japan; 2 Laboratory of Biomaterials, Institute for Frontier Life and Medical Sciences, Kyoto University, 53 Kawara-cho Shogoin, Sakyo-ku, Kyoto, 606-8507 Japan

**Keywords:** Rotator cuff tear, Basic fibroblast growth factor (bFGF), Platelet-rich plasma (PRP), Rotator cuff repair, Rat model, Infraspinatus tendon

## Abstract

**Introduction:**

Excellent outcomes of arthroscopic rotator cuff repair for small and medium tears have been recently reported. However, re-tears after surgery have been a common complication after surgical repair of large and massive rotator cuff tears and often occur in early postoperative phase. It was previously reported that basic fibroblast growth factor and platelet-rich plasma enhanced rotator cuff tear healing. We hypothesized that this combined therapy could enhance rotator cuff healing after rotator cuff repair in a rat model. This study aimed to evaluate the efficacy of combined therapy of platelet-rich plasma and basic fibroblast growth factor with gelatin-hydrogel sheet.

**Methods:**

To create a rotator cuff defect, the infraspinatus tendon of Sprague Dawley rat was resected from the greater tuberosity. The infraspinatus tendons were repaired and covered with gelatin-hydrogel sheet impregnated with PBS (control group), basic fibroblast growth factor (bFGF group), platelet-rich plasma (PRP group), or both basic fibroblast growth factor and platelet-rich plasma (combined group). Histological examinations were conducted using hematoxylin and eosin, safranin O, and immunofluorescence staining, such as Isolectin B4, type II collagen at 2 weeks postoperatively. For mechanical analysis, ultimate failure load of the tendon-humeral head complex was evaluated at 6 weeks postoperatively.

**Results:**

In the hematoxylin and eosin staining, the tendon maturing score of the combined group was higher than that of the control group at postoperative 2 weeks. In the safranin O staining, stronger proteoglycan staining was observed in the combined group compared with the other groups at postoperative 2 weeks. Vascular staining with isolectin B4 in 3 treatment groups was significantly higher than that in the control group. Type II collagen expression in the combined group was significantly higher than those in the other groups. The ultimate failure load of the combined group was significantly higher than that of the control group.

**Conclusion:**

Combined therapy of basic fibroblast growth factor and platelet-rich plasma promoted angiogenesis, tendon maturing and fibrocartilage regeneration at the enthesis, which could enhance the mechanical strength. It was suggested that combined basic fibroblast growth factor and platelet-rich plasma might enhance both tendon and bone–tendon junction healing, and basic fibroblast growth factor and platelet-rich plasma might be synergistic.

## Introduction

Rotator cuff tear is common in the elderly population and the outcome of surgical treatment using arthroscopic rotator cuff repair for small cuff tear is satisfactory [1, 2]. In contrast, surgical treatment of large and massive cuff tear is still challenging because of re-tear after surgery. Rotator cuff re-tear is a common complication after surgery that usually occurs in early postoperative phase [3, 4]. The re-tear ratio is reportedly 14–66% and re-tear can lead to loss of shoulder motion and shoulder function, and recurrence of pain [4–8]. To prevent re-tear after surgery, enhancement of biological healing and enforcement of mechanical strength at the repaired site could be required. There are some reports of reconstruction using artificial materials, such as Teflon, but the foreign body reaction became a problem because it remained as a non-biomaterial in the body [9]. Moreover, there are other reports of artificially absorbable materials, such as PLLA (polylactic acid) [10], PGA (polyglycolic acid) [11], PLG (polylactic acid glycolic acid) [12], and cell sheets derived from human rotator cuff [13]. Footprint augmentation with fascia lata graft is clinically reported for large and massive rotator cuff tears [14]. Using fascia lata graft, collagen expression enhancement was observed at the repaired site. Compared with single-row repair, better mechanical property in fascia lata grafting is also reported [15]. However, harvesting fascia lata can cause donor site morbidity, such as pain, hematoma formation, and scar formation. The use of tissue engineering technique is an attractive tool to solve this problem. There are several growth factors that could enhance the tendon healing process [16]. Basic fibroblast growth factor (bFGF) expression is reported to be upregulated at healing site 1 week after supraspinatus repair [17, 18]. It was reported that Platelet-rich plasma (PRP) also enhanced the healing process after rotator cuff repair in a mouse model [19]. These investigations were performed by a single administration of growth factors without any carriers. Considering the drug-delivery system, carrier of these growth factors could be important for better effect of drug. Gelatin is a biodegradable polymer extensively used for medical purposes and its biosafety and biocompatibility have been demonstrated through long clinical applications and a number of tissue engineering studies [20–24]. Gelatin-hydrogel combined with growth factor successfully enhanced the tissue regeneration of fracture, ligament injury, and fibrocartilage [25, 26]. Matsui et al. reported that PRP or bFGF or mixed PRP and bFGF was administered to a mouse leg ischemia model using the gelatin-hydrogel granules, and angiogenesis was more strongly confirmed in the mixed group [27]. We speculated that controlled release of bFGF and PRP might enhance the regeneration of repaired site, as growth factors can affect the surrounding tissue in a longer period. In this study, gelatin-hydrogel sheet (GHS) with/without bFGF and PRP is transplanted to the rotator cuff repaired site in a rat rotator cuff injury model. We hypothesized that this combined therapy could enhance rotator cuff healing after rotator cuff repair in a rat model. This study aimed to evaluate the efficacy of combined therapy of platelet-rich plasma and basic fibroblast growth factor with gelatin-hydrogel sheet.

## Materials and methods

All animal experiments were approved by the committee of our institute.

### PRP preparation

PRP was prepared by double-spin method [27]. Sprague Dawley (SD) rats were anesthetized with isoflurane (Wako, Tokyo, Japan) and intraperitoneal injection of pentobarbital sodium (50 mg/kg; Kyoritsu Seiyaku, Tokyo, Japan). Briefly, rat blood (10 ml) was collected into tubes containing acid-citrate-dextrose solution and centrifuged for 7 min at 450 g and 4 °C. Next, the yellow plasma with buffy coat was centrifuged for 5 min at 1600 g and 4 °C. The platelet pellet was collected and the thrombolytic pellet in 1 ml of plasma was used as PRP. The density of platelets in the *PRP:* PRP prepared was increased by a factor of 5 when compared with that of the original blood (1.5–2.0 * 10^8^/ml plasma). To activate PRP for the release of growth factors, the PRP preparation was mixed with CaCl2 solution at concentrations of 2 wt.% at a ratio of 7:1 by volume and then left for 1 h at 37 °C.

### bFGF

An aqueous solution of human recombinant bFGF (Kaken Pharmaceutical Co., Ltd., Tokyo, Japan) was diluted with physiological saline solution (Otsuka Pharmaceutical Co., Ltd., Tokyo, Japan) to give a solution concentration of 500 µg/ml. Tokunaga et al. reported that this dose of bFGF promotes growth of the tenogenic progenitor cells, resulting in biomechanical and histological improvement of the repaired rotator cuff of rats [18]. We determined the bFGF concentration according to this report.

### GHS preparation

Pigskin gelatin with a molecular weight of 100,000 Da and an isoelectric point of 5.0 was supplied by Nitta Gelatin Co., Ltd. (Osaka, Japan). After preparing with 5 wt% aqueous solution of gelatin-hydrogel, the solution was cast into polystyrene dish as thinly as possible and frozen at -80 °C in deep freezer. After the solution was frozen, freeze-drying was performed for 48 h. Freeze-drying gelatin-hydrogel was cut into 2 mm × 2 mm segments. The segments were cross-linked by dehydrothermal treatment at 140 °C for 48 h in a vacuum oven [28].

A solution of 5 ul of PRP or bFGF was dropped onto a GHS for impregnation. Similarly, empty gelatin hydrogels without bFGF were prepared by adding PBS to the solution. Finally, 4 kinds of GHS were created according to experimental groups as PBS (control), PRP, bFGF, and combined groups (PRP and bFGF). The release kinetics have been reported by Matsui et al., and both bFGF alone and PRP plus bFGF have shown sustained release of bFGF. It was gradually released immediately after entering the living body, and it was released for about 3 weeks. During that time, the activity was maintained [27].

### GHS transplantation

**Fig. 1:**
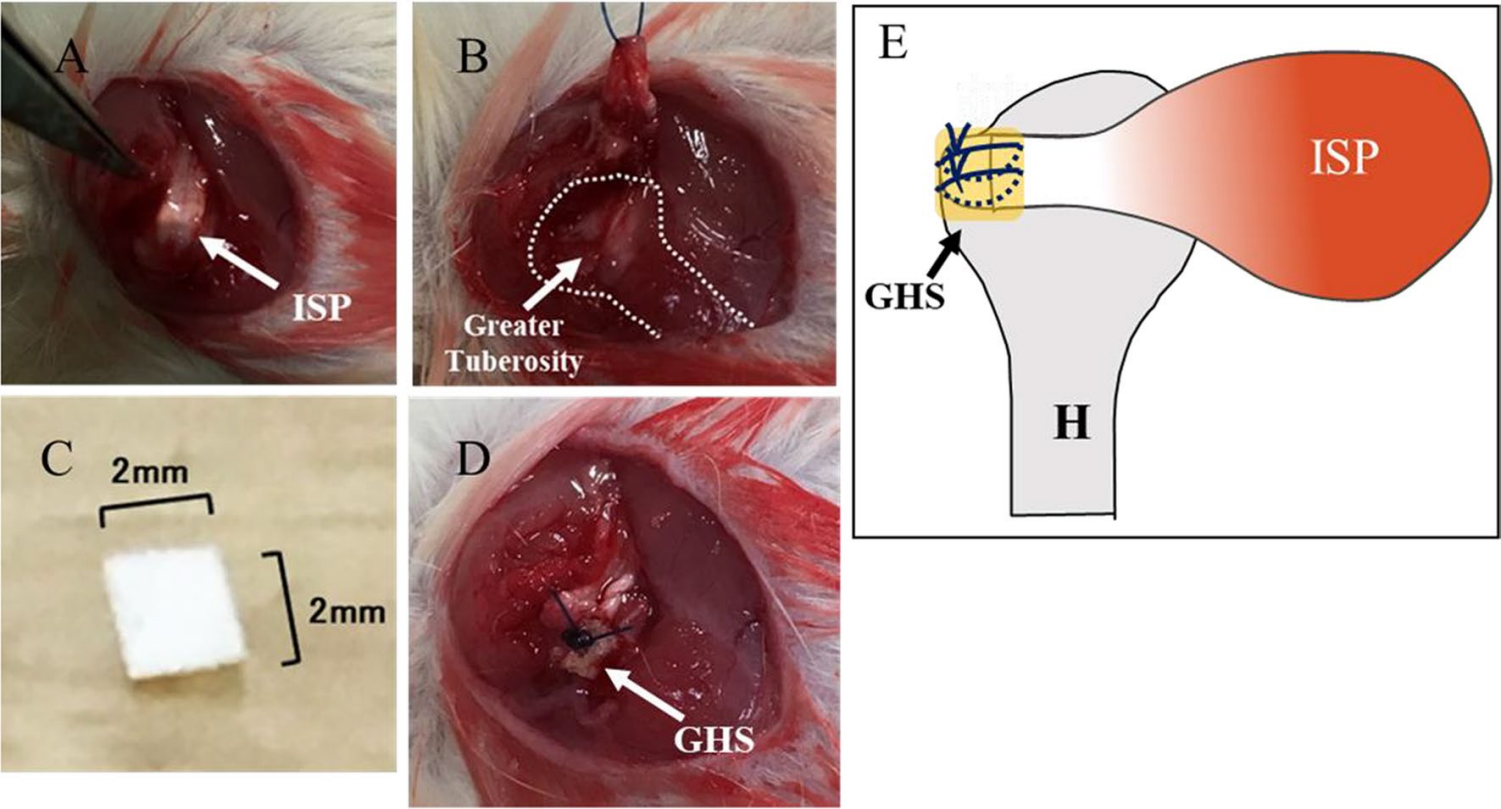
**A** Infraspinatus tendon was identified. **B** Infraspinatus tendon was cut off at the insertion to the greater tuberosity. **C** Gelatin-hydrogel sheet (GHS). **D** Infraspinatus tendon was repaired by a transosseous technique, and the repaired site was covered with GHS. **E** Scheme of ISP (infraspinatus tendon) repair with GHS. Yellow square is transplanted GHS. (H, humeral head)

In this study, 40 SD rats (12-week-old) with a mean weight of 250 g (CLEA Japan, Inc., Tokyo, Japan) were used. Forty rats were divided into 4 groups of 10 each. All operations were performed under sterile conditions and anesthesia with isoflurane (Wako), intraperitoneal injection of pentobarbital sodium (50 mg/kg; Kyoritsu Seiyaku), and subcutaneous injection of lidocaine (2.5 mg/kg, Xylocaine®; AstraZeneca, London, UK) at the surgical site. The animals were placed in a lateral position, and a 1 cm incision was made over the lateral border of the acromion. A small portion of the deltoid muscle was divided to expose the underlying acromion and the infraspinatus tendon. The infraspinatus tendon was carefully identified (Fig. 1A) and cut off at the insertion to the greater tuberosity (Fig. 1B). The footprint was abraded to remove normal enthesis with a high-speed bur until bleeding was observed. In the right shoulders, tendons were repaired by a transosseous technique using 4–0 nylon suture, and the repaired site was covered with GHS (Fig. 1C, D). Four kinds of GHS were transplanted in each group (n = 10 each group). The deltoid and skin were closed with 4–0 nylon. After transplantation, all rats were immediately allowed to move freely within their own cage in laminar flow rack. All rats were actively moving. Rats were euthanized with overdose of isoflurane and intraperitoneal injection of pentobarbital sodium at the indicated times.

#### Histological examination

For the histological examination, 6 rats from each group were sacrificed at 2 weeks after transplantation. The scapular-humeral complexes were harvested and quickly embedded in optimal cutting temperature compound (Sakura Finetek USA, Inc., Torrance, CA) and stored at − 80 °C for histochemical and immunohistochemical staining as described below. Tissue sections were stained with hematoxylin and eosin (H&E) and safranin O for histological characterization of tissue composition, and the histological findings were evaluated at 2 points: the tendon proper and tendon insertion using light microscopy. Watkins et al. reported the tendon maturing scoring system to quantitatively evaluate the regenerated tendon [29]. Six histological parameters, such as cellularity, fibrocytes, vascularity, fiber diameter, parallel cells, and parallel fibers, were evaluated to identify the characteristics of the maturity of cellular and intercellular constituents. In the safranin O staining, proteoglycan content was calculated as a percentage of the pixels of each tendon–bone interface (positive/total pixels) using Adobe Photoshop CC 2015 software (Adobe Systems Incorporated, San Jose, USA). In immunofluorescence staining, isolectin B4 antibody (Vector Laboratories, Burlingame, CA), a rat-specific endothelial marker, was used to access the regenerated capillaries and neovascularity. Type II collagen (Col2) antibody (Cosmo Bio Co., Ltd., Tokyo, Japan) was used to assess fibrocartilage regeneration. Antibodies were used at a 1:100 dilution, and staining was performed at room temperature for 1 h. DAPI solution was applied for 5 min for nuclear staining. After staining, we evaluated the number of positively stained cells in 5 randomly selected fields. Histological examination was performed blindly by two examiners.

### Mechanical analysis

For mechanical analysis, ultimate failure load of the tendon-humeral head complex was evaluated at 6 weeks postoperatively.

Six weeks after surgery, 4 rats from each group were euthanized, and shoulders were biomechanically tested. All soft tissues except the infraspinatus tendon-humeral complex were carefully removed before the biomechanical tests. The prepared infraspinatus-humeral complex was mounted in a conventional tensile tester (model AGIS 5kN; Shimadzu, Kyoto, Japan) [13]. The humerus was embedded in an aluminum tube using polymethylmethacrylate. The proximal end of the infraspinatus tendon was glued between two pieces of sandpaper. Testing was performed with the shoulder at 60° of abduction in a testing machine. The humerus was clamped with its long axis in the horizontal plane. The proximal end of the infraspinatus tendon was glued between 2 pieces of sandpaper. The sandpaper-tendon complex was clamped vertically. The biomechanical testing protocol that we used was similar to that described by Galatz et al. [30] and Mikolyzk et al. [31] Specimens were subjected to a preload of 0.2 N and were preconditioned for 5 cycles to 0.38 mm of displacement (approximately 5% of gage length at a rate of 0.1 mm/s). A stress relaxation test was then performed for 300 s at 0.38 mm of displacement followed by 300 s of recovery. Specimens were then tested to failure in tension at a rate of 0.1 mm/s. The ultimate failure load was determined for each specimen.

### Statistical analysis

All data are expressed as mean values ± standard deviations. One-way ANOVA analysis followed by Tukey–Kramer analysis was performed for comparison of 4 groups. *P* < 0.05 was considered statistically significant. SPSS (version 23.0; IBM Corporation, Armonk, NY) was used for data analysis.

## Results

### Histological analysis

**Fig. 2:**
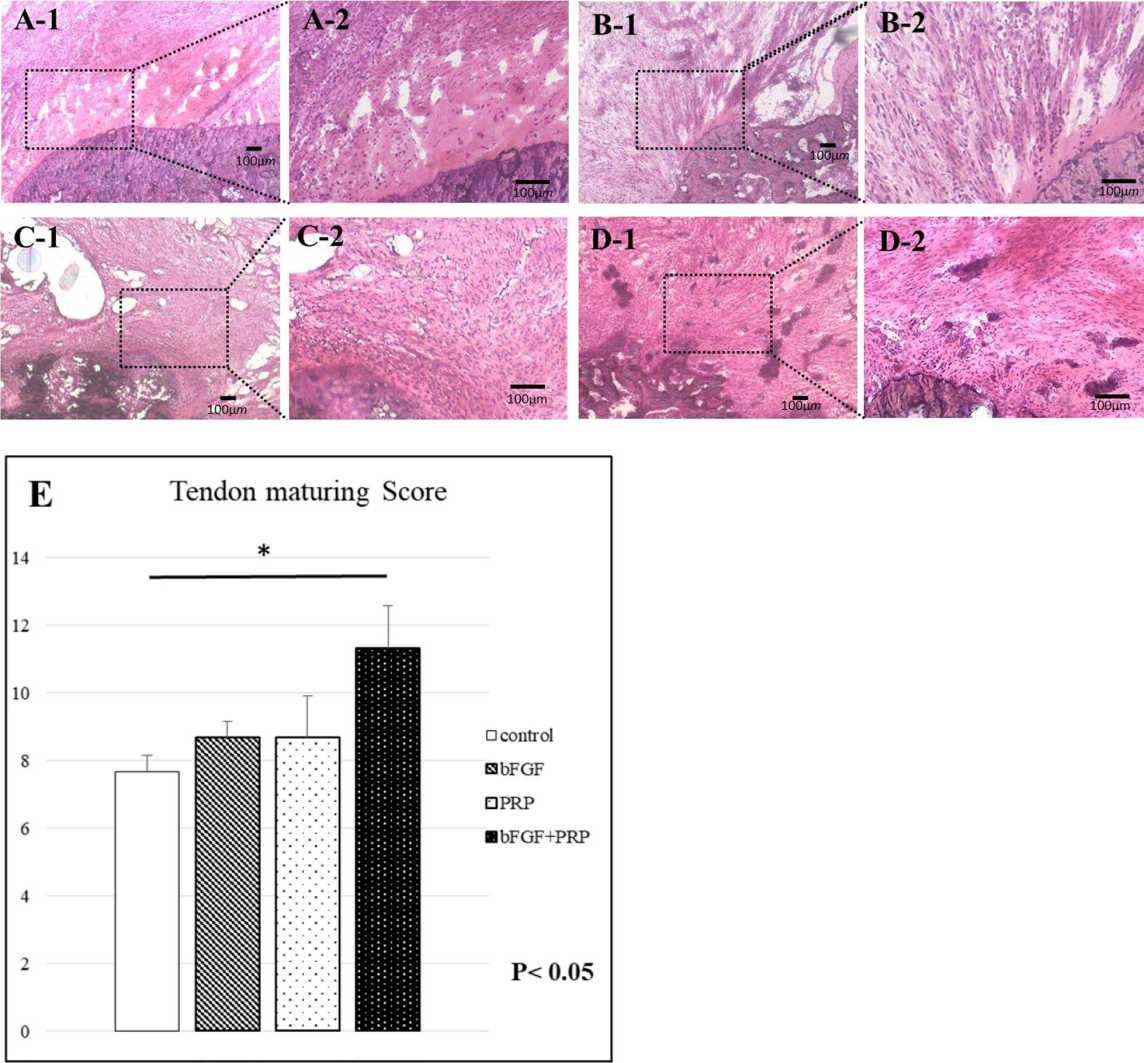
H&E staining at 2 weeks postoperatively, **A-1,2**: control group, **B-1,2**: bFGF group, **C-1,2**: PRP group, **D-1,2**: bFGF + PRP combined group). **E**: Tendon maturing score. The tendon maturing score of the combined group was higher than the control group

**Fig. 3:**
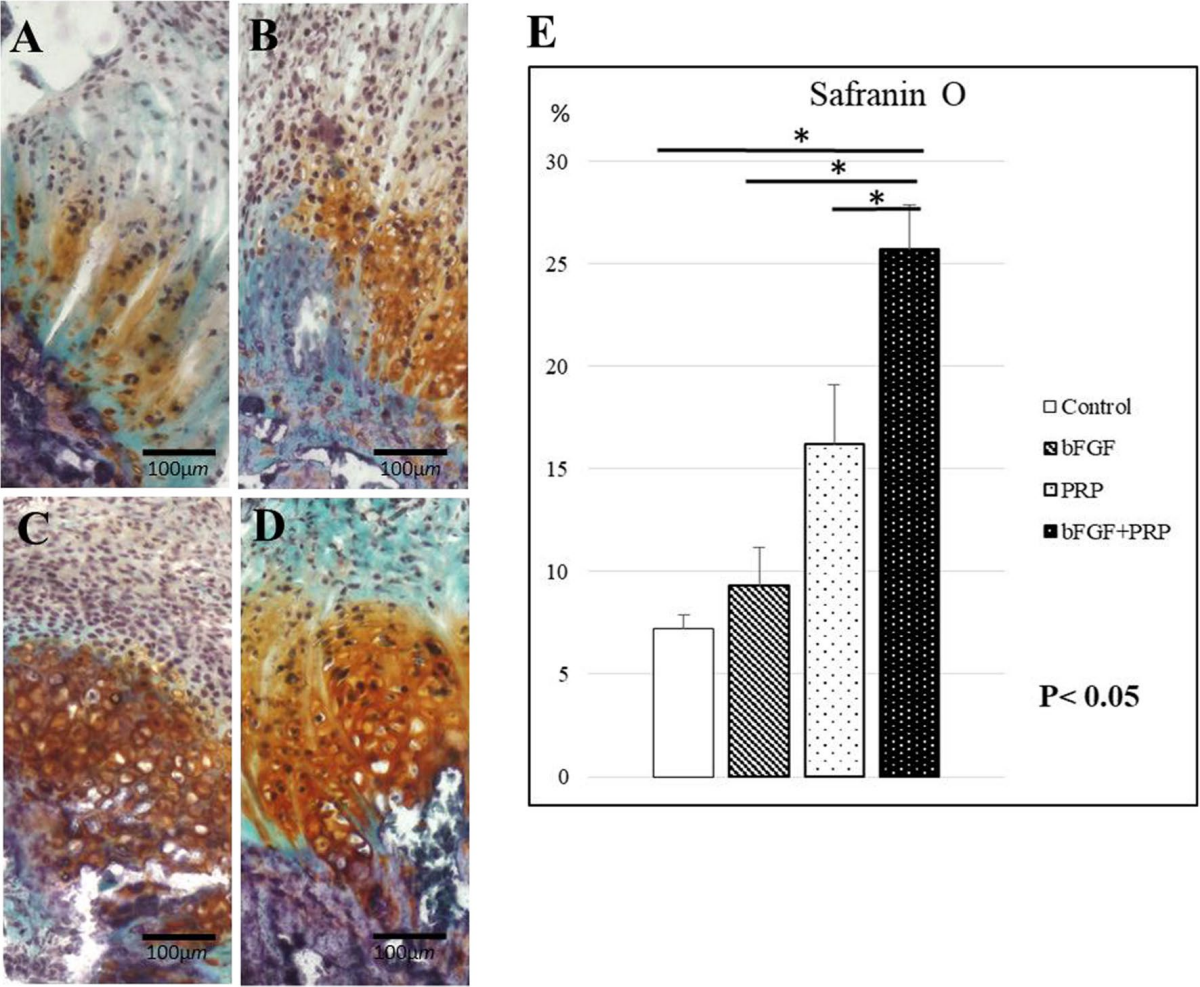
Safranin O staining at 2 weeks postoperatively, **A**: control group, **B**: bFGF group, **C**: PRP group, **D**: bFGF + PRP combined group. **E**: Proteoglycan content was calculated as a percentage of the pixels of each tendon–bone interface (positive/total pixels). Significantly stronger proteoglycan staining was observed at the repaired enthesis in combined group compared with other groups

**Fig. 4:**
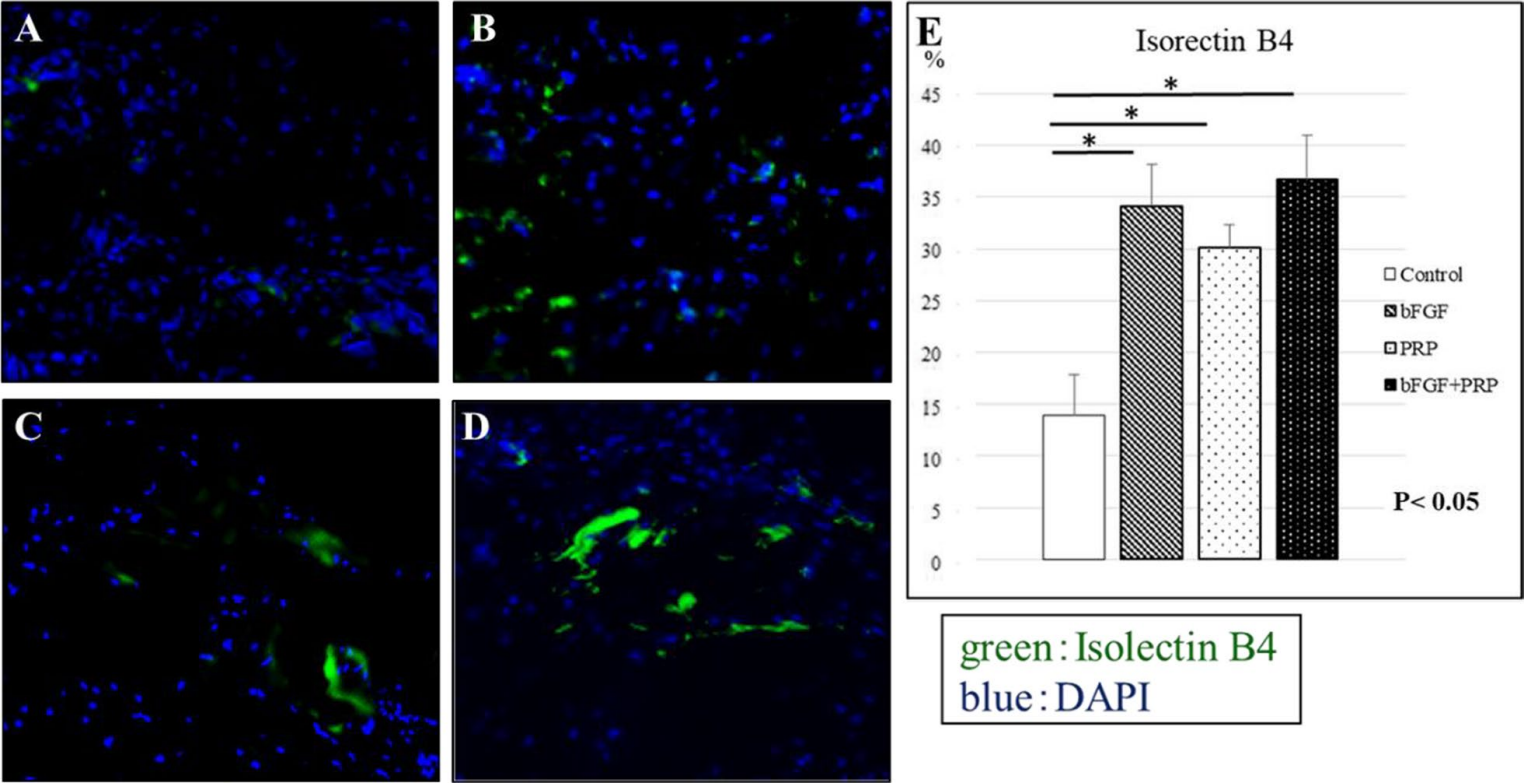
Immunofluorescence staining: isolectin B4 at 2 weeks postoperatively (**A**: control group, **B**: bFGF group, **C**: PRP group, **D**: bFGF + PRP combined group). **E**: The number of positively stained cells in 5 randomly selected fields. Vascular staining with isolectin B4 in treatment groups was significantly higher than that in the control group

**Fig. 5:**
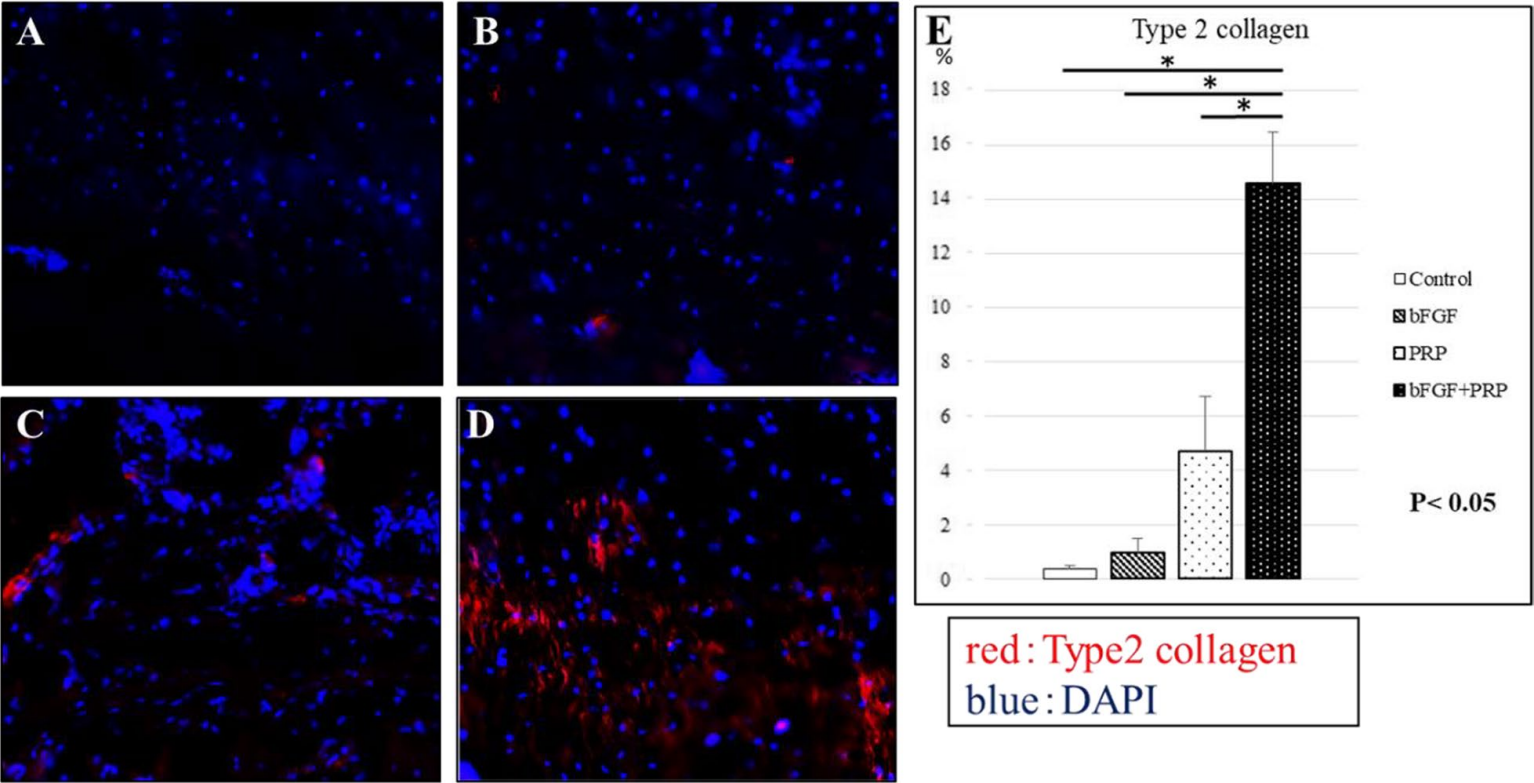
Immunofluorescence staining: type II collagen at 2 weeks postoperatively (**A**: control group, **B**: bFGF group, **C**: PRP group, **D**: bFGF + PRP combined group). **E**: The number of positively stained cells in 5 randomly selected fields. Type II collagen in the combined group was significantly higher than that in other groups

In H&E staining, tendon maturing scores in control, bFGF, PRP, and combined groups were 67 ± 0.47, 8.67 ± 0.47, 8.67 ± 1.25, and 11.3 ± 1.25, respectively (Fig. 2). The score in the combined group was significantly higher than that in the control group at postoperative 2 weeks (*p* < 0.05). The quantitative analysis with safranin O staining at the tendon–bone junction showed scores of 7.2 ± 0.69%, 9.3 ± 1.9%, 16.2 ± 2.9%, and 25.7 ± 2.2% in control, bFGF, PRP, and combined groups, respectively (Fig. 3). The significantly stronger proteoglycan staining was observed at the repaired enthesis in the combined group compared with the other groups at postoperative 2 weeks (*p* < 0.05). The percentages of positive cells in vascular staining with isolectin B4 in control, bFGF, PRP, and combined groups were 1.4 ± 0.38%, 3.4 ± 0.41%, 3.0 ± 0.22%, and 3.7 ± 0.43%, respectively (Fig. 4). The 3 groups with the growth factors showed significantly higher vascular expression than in the control group (*p* < 0.05). Finally, Col2 expression in control, bFGF, PRP, and combined groups were 0.34 ± 0.12%, 0.95 ± 0.53%, 4.7 ± 2.0%, and 14.6 ± 1.9%, respectively (Fig. 5). The combined group showed significantly higher expression than those in the other groups (*p* < 0.05).

### Mechanical analysis

**Fig. 6:**
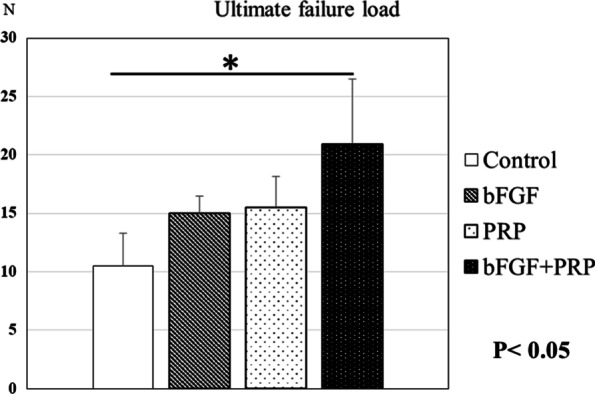
The ultimate failure load of the combined group was significantly higher than that of the control group at 6 weeks after surgery

The ultimate failure load in control, bFGF, PRP, and combined groups was 10.5 ± 2.8 N, 15.0 ± 1.4 N, 15.5 ± 2.6 N, and 21.0 ± 5.5 N, respectively (Fig. 6). The combined group showed significantly higher failure load than that of the other groups (*p* < 0.05).

## Discussion

There were many laboratory experiments that used growth factors for tendon healing. Local administration of bFGF enhanced tendon–bone interface healing [17]. Tokunaga et al. reported that the use of bFGF stimulates the proliferation of tenogenic progenitor cells leading to higher expression of tenogenic markers in a rat model [18]. PRP contains various growth factors, such as platelet-derived growth factor-B, transforming growth factorβ-1, vascular endothelial growth factor, and epithelial growth factor [32]. After PRP activation, the platelets in PRP release various growth factors through the degranulation of α-granules. Then, the growth factors exhibit various biological activities. It was reported that PRP administration into the articular cartilage defect enhanced cartilage regeneration [33]. Release of platelet-derived factors directly at the site of cartilage damage can stimulate a natural healing cascade and tissue regeneration. The release of various growth factors from its alpha granules of activated platelet, promoting cartilage substrate synthesis and promoting protein transcription within chondrocytes. Chemical attractants stored in platelets attract proteins, such as fibrin, which act as the first scaffold for stem cells to migrate and differentiate. The positive effect of PRP after rotator cuff repair in a rat model has also been reported [34]. In spite of good results with growth factors in laboratory study, clinical situation might differ because dose and healing duration is different in animal experiments. The drug-delivery system of growth factors is considered as a key to improve tissue regeneration. Once the growth factor in solution form is injected, biological activity is not always expected because the growth factor activity is unstable due to rapid enzymatic digestion or deactivation. Intra-articular injection of the growth factor solution can be easily diluted after shoulder joint surgery. Therefore, drug-delivery system of growth factors is essential to efficiently improve biological functions. The use of GHS has been successful in the controlled release of various growth factors and PRP [35]. The released growth factors were impregnated into gelatin-hydrogel sheet through various intermolecular interactions, such as electrostatic and hydrophobic interactions. bFGF with an IEP of 9.6 was well sorbed to the acidic gelatin hydrogel through ionic interaction. Gelatin-hydrogel sheet was enzymatically degraded in vivo to produce water-soluble gelatin fragments, so this factor was continuously released around the hydrogels until degradation of GHS occurs. This GHS was degraded for 2 weeks, and sustained release of the encapsulated growth factors occurred [27]. Kabuto et al. compared intra-articular injection of Bone Morphogenetic Protein-7 (BMP-7) and GHS impregnated with BMP-7 in a rat rotator cuff repair model [36]. Slow release of BMP-7 was observed in GHS group up to 3 weeks after surgery yielding better tissue regeneration. In this study, we used bFGF and PRP as growth factors for rotator cuff regeneration. Among the various kinds of growth factors, we chose these 2 factors as these are approved growth factors for clinical use in our country. Administration of single factor with GHS showed better histological outcomes compared with the control group. Moreover, use of both factors showed the best histological and mechanical properties. In a study of mouse ischemic limb, dual release of PRP and bFGF impregnated in the biodegradable gelatin-hydrogel granules promoted not only angiogenesis but also maturation of blood vessels [27]. In this study, combined bFGF and PRP therapy promoted angiogenesis at the tendon–bone interface, which might lead to better tendon and fibrocartilage regeneration compared with the control. Basic fibroblast growth factor and platelet-rich plasma may synergistically promote tendon–bone–tendon interface healing and provide initial strength for rotator cuff repair. In the future, it will be necessary to translate this result into clinical practice. First, we need to proceed with the process of applying it to human testing and research development [37].

This study has several limitations. First, this rat model was an acute rotator cuff injury model. The animal models may differ from chronic elderly human rotator cuff injury. Second, the anatomy between the rat shoulder and that of humans are different, and the short rotator cuff muscles of rat do not form a rotator cuff that is similar to humans. Third, rats have greater healing capacity than humans; hence the tendon–bone healing process progressed faster than that in humans. The negative points of the rodent model are that the rat model may not be directly converted to humans, as the healing process of healthy animals may not reflect the circumstances encountered in clinical practice. However, this rat model is widely used to investigate healing mechanisms and ways to promote healing. The fourth, in this study, the small sample size of each group limited the ability to detect significant differences. The fifth, there were few time points and the period was short. Finally, the dose of growth factors and the kinetics was determined according to the previous report and we experimented with only a single dose and did not optimize its dose as growth factor composition in PRP might differ from patient to patient. There were individual differences in PRP, and not all of them contained the same amount of growth factors, so it was necessary to classify and evaluate PRP, but this time we had not done so.

## Conclusion

Combined bFGF and PRP therapy promoted angiogenesis, tendon maturing and fibrocartilage regeneration at the enthesis and the mechanical strength. PRP and bFGF enhance both tendon and bone–tendon junction healing, and b-FGF and PRP might be synergistic. The combined therapy of PRP and bFGF using GHS could synergistically work and enhance rotator cuff healing after rotator cuff repair.

## Data Availability

Not applicable. All data are presented in the manuscript.

## Abbreviations

PBS: Phosphate buffered saline
PRP: Platelet-rich plasma
bFGF: Basic fibroblast growth factor
GHS: Gelatin-hydrogel sheet
BMP-7: Bone morphogenetic protein-7
